# Clinical and Genetic Analysis of a Nonsyndromic Oligodontia in a Child

**DOI:** 10.1155/2014/137621

**Published:** 2014-08-25

**Authors:** Orlando Lopes Coelho Neto, Maria Fernanda Reis, Ticiana Medeiros de Sabóia, Patrícia Nivoloni Tannure, Leonardo Santos Antunes, Andréa Gonçalves Antonio

**Affiliations:** ^1^School of Dentistry, Universidade Veiga de Almeida, 20271-020 Rio de Janeiro, RJ, Brazil; ^2^Clinical Research Unit, Universidade Federal Fluminense, 20271-020 Niterói, RJ, Brazil; ^3^Department of Pediatric Dentistry and Orthodontics, School of Dentistry, Universidade Federal do Rio de Janeiro, 21941-902 Rio de Janeiro, RJ, Brazil; ^4^Department of Specific Formation, School of Dentistry, Universidade Federal Fluminense, 28625-650 Nova Friburgo, RJ, Brazil

## Abstract

The etiology of tooth agenesis may be related to several factors, among them, the genetic alterations that play a fundamental role in the development of this dental anomaly, so that knowledge about it helps the clinician to have a greater understanding of their patients. Thus, the aim of this study was to report the case of a nonsyndromic child, with tooth agenesis of one premolar, three first permanent molars, and all second permanent molars. In addition, a genetic research between polymorphic variants in genes MMP3 and BMP2 was performed in order to observe the association between changes in these genes and congenital tooth absences. For this purpose, DNA from child was extracted and polymorphisms were investigated. It was clinically and radiographically observed that this was a case of oligodontia, in which the authors suggested an association between the polymorphisms found and tooth agenesis diagnosed in that child.

## 1. Introduction

Tooth agenesis may be defined as the congenital absence of one or more teeth, with the exception of third molars, with this being one of the most prevalent dental developmental anomalies, with rates ranging from 3.2% to 13.3% in different populations [1]. When the number of absent teeth exceeds the value of 6 elements, the term used for cases of congenital absences is oligodontia, while anodontia represents the complete congenital absence of teeth [2]. The etiology of congenital tooth absence may be related to genetic, nutritional, traumatic, infectious, and hereditary factors, with the latter being considered the main etiologic factor of this condition [3]. According to Lexner et al. [4], tooth agenesis is frequently found in individual with genetic syndromes or disorders or in fissure patients, or it may even occur as an isolated case [2, 5].

When considering isolated cases or the nonsyndromic form, many studies have investigated the correlation between tooth agenesis and genetic mutations present in individuals belonging to one and the same family, attributing the mutations to genes PAX9, MSX1, AXIN2, and EDA [6–9]. However, many oligodontia families reported in the literature could not be identified as having any mutations in these genes [10, 11]. According to Küchler et al. [12], variations in genes that are critical for tooth formation may contribute to the tooth agenesis. In a recent study [13], the authors affirmed that polymorphism in BMP2 contributed to isolated cases of tooth agenesis. Also, MMPs are potential candidate genes for dental alterations based on the roles they play during embryogenesis [12]. Letra et al. [14] observed an association between a MMP3 polymorphism and cleft lip and/or palate.

Since lip, palate, and tooth development are influenced by the same genes, the aim of the present study was to report the case of a child with oligodontia, in whom an investigation between the polymorphic variants in genes MMP3 and BMP2 was performed.

## 2. Case Report

A 9-year-old girl, leukoderma, presented at the Pediatric Dental Clinic of “Universidade Veiga de Almeida,” accompanied by her guardian, with the complaint of misaligned teeth. A term of consent was obtained, authorizing the present case report to be made. Her medical history revealed a normal birth, presenting absence of systemic compromise or any other relevant datum. On extraoral clinical exam as well, no alteration was observed. Considering the dental history, it was revealed that the child had already been submitted to dental appointments only to have prophylaxis and fluoride application.

**Figure 1:**
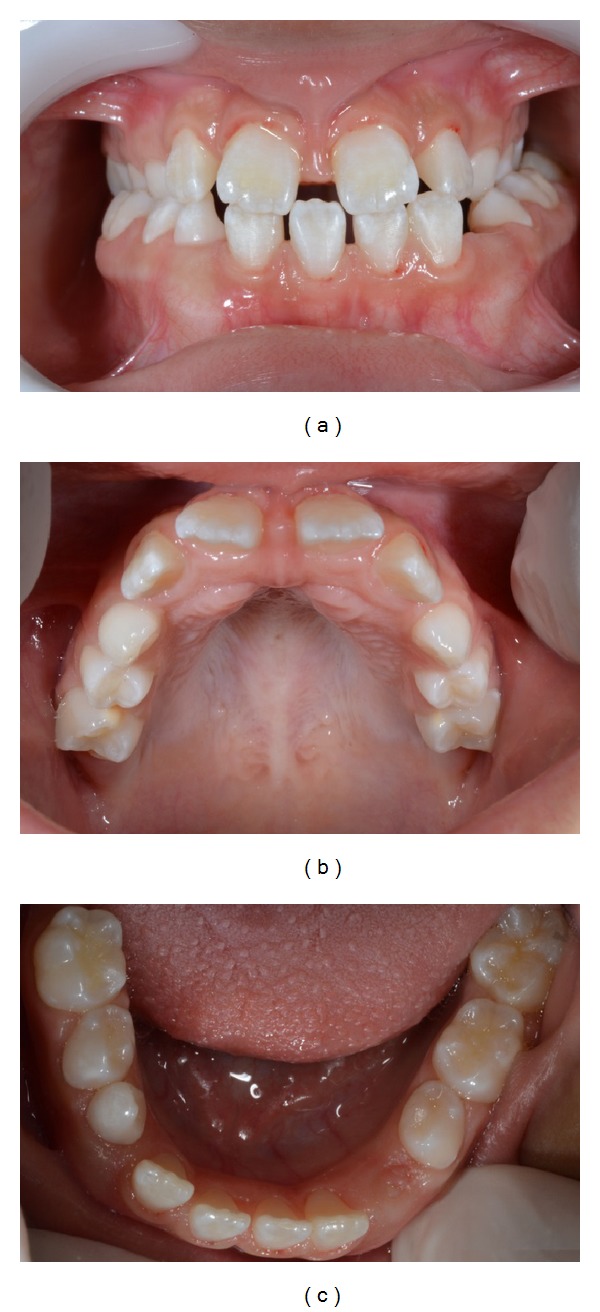
Intraoral photographs. (a) Front view; (b) maxillary arch; (c) mandibular arch.

**Figure 2:**
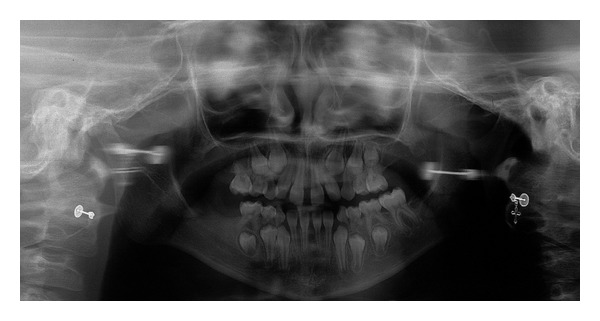
Panoramic radiograph demonstrating absence of teeth.

**Figure 3:**
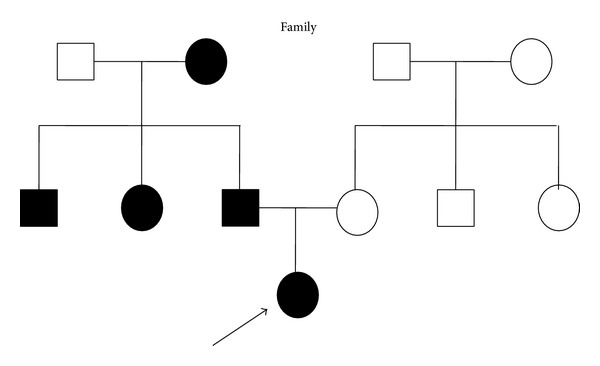
Pedigrees of the oligodontia family with arrows indicating the proband. Black figures = affected; open figures = unaffected; squares = males; circles = females.

After meticulous intraoral clinical exam, it was found that the child was at the stage of mixed dentition, with absence of three first permanent molars, deviation from the midline, bilateral posterior crossbite, and deep palate (Figure 1). After radiographic exam, the following tooth absences were confirmed: elements 16, 26, and 46 and the tooth germs of element 15 and all the permanent second molars (Figure 2). According to her guardian's report, other members of the family presented tooth agenesis, such as the patient's paternal grandmother, uncle, and aunt, as well as her father (Figure 3).

It is worth adding that the patient was referred for orthodontic treatment, and at the appropriate age, after correcting the bone and tooth discrepancies, she will undergo prosthetic rehabilitation. In addition, follow-up consultations will be scheduled regularly for oral hygiene instructions.

## 3. Genetic Analysis

Epithelial cells from the oral mucosa were collected as a source of genomic DNA, following a procedure based on a previously published protocol [17]. Only the DNA sample with a ratio of 260/280 above 1.8 was analyzed. Two markers in the 2 genes (Table 1) were genotyped by polymerase chain-reactions with the TaqMan method [18], with specific probes for allelic distinction, performed with the Stratagene Mx3005P real-time PCR system (Stratagene, La Jolla, CA, USA). Predesigned probes were supplied by Applied Biosystems (Foster City, CA, USA).

## 4. Results of Selection of Candidate Genes and Single Nucleotide Polymorphisms

We selected genes involved in craniofacial development and a polymorphism (rs522616) that was previously investigated in a Brazilian population affected by oral clefts. According to Letra et al. [15] a genetic marker in MMP3 (rs522616) showed significant association with all cleft types in individuals from Brazil. In addition, the ancestral allele (A) in this polymorphism was associated with increased risk of oral clefts. Our genetic analysis found the genotype AA for the patient analyzed. In other words, the child carried two copies of the A allele associated with oral clefts.

Considering gene BMP2, a recent study [13] identified this gene with a role in skeletal development. Craniosynostosis (CS), the premature closure of one or more of the cranial vault sutures, is a common congenital anomaly. According to Justice et al. [13] the same polymorphism studied here (rs1884302) was considered the most significant SNP for this malformation. The minor allele C was associated with the disease. Our analysis revealed the CC genotype. We hypothesized that the child carries two copies of the C allele, the minor allele probably being associated with a congenital malformation.

## 5. Discussion

Candidate genes, such as PAX9, MSX1, AXIN2, and EDA [6–9, 19–24], are related to etiology of tooth agenesis. However, other genes are being investigated for the same condition. Among them, the matrix metalloproteinase genes (MMPs) are outstanding, due to the important function of these enzymes during craniofacial development [25] and also the BMPs (bone morphogenetic protein genes) that participate directly in the cascades of events during the initial stages of odontogenesis [12]. In the present case report, the polymorphisms rs522616 (MMP3) [15] and rs1884304 (BMP2) [12], which are functional polymorphisms, were selected. Letra et al. [15] affirmed that although the exact function has not been elucidated the variant rs522616 (MMP3) is located in the gene promoter and has regulatory effect on gene transcription and function. A recent study [16] suggests that the A allele can enhance promoter activity, possible augmenting transcription factor binding. Also, Mu et al. [8], using bioinformatics, found that a BMP2 polymorphism exhibited different BMP2 mRNA structures, and the G allele required more energy for mRNA secondary structure stabilization than the A allele did.

The functional SNPs were chosen due to the fact of being capable of influencing genic expression, increasing or diminishing the final quantity of protein coded by that gene [26]. A recent study [27] also suggested that gene BMP2 is strongly associated with cleft lips and that cases of absent teeth are directly related to the severity of the cleft found in the patient [28]. In the same way, Küchler et al. [12] affirmed that polymorphisms in gene MMP3 were also associated with cleft lips and palates and this evidence came from studies that also showed association with tooth agenesis [15]. Bartzela et al. [29] also stated that the frequency of dental anomalies seems to be linked to the severity of the cleft malformation. After performing the exams of the child in this report, no type of cleft palate was found.

Apart from the literature cited, it is important to stress the point that dental anomalies including tooth agenesis are subclinical phenotypes (mild forms) of over clefts mainly observed in unaffected relatives [14]. It is worth adding that, in spite of the genetic investigations of the present case having demonstrated polymorphisms in both gene MMP3 and BMP2, these results must be interpreted with caution, because it is an isolated case in which the genetic evaluation was not performed in the entire family. For this reason, previous studies [13, 15, 16] in the literature were consulted so that a possible association between the alterations verified and the ageneses could be established. An extensive search of the literature was conducted, but no other study was found with polymorphisms in the studied genes and patients with tooth ageneses.

The diagnosis of tooth agenesis is dependent on the anamnesis and clinical and radiographic exams being performed with acuity by the dentist. Moreover, in the early diagnosis of tooth agenesis, it has become imperative to use panoramic radiographs. This exam was requested in order to make the diagnosis of the tooth absences in the present case, in which ageneses of the following permanent teeth were found: maxillary left second premolar, maxillary right and left first molars, mandibular right first molar, and all second molars. The prevalence of absence of second premolars, lateral incisors [30], and third molars [7] was remarkable. However, ageneses of first and second molars, as in the present report, are rare conditions. According to Abe et al. [31], the prevalence of absence of maxillary first molars is 0.5%.

According to the literature [31], there is a high prevalence of ageneses of permanent first molars in a symmetrical manner in the maxillary arch. However, nothing was found with respect to the bilateralism of absences of this same tooth in the mandibular arch. The child investigated in this study presented symmetrical or bilateral absences of permanent first molars in the maxilla, whereas, in the mandible, there is agenesis only of the permanent right first molar.

When we consider the ageneses of the permanent second molar germs, we should take into consideration that Moyers [32] reported the beginning of development of the permanent second molars at 3 years of age for both genders. Therefore, at 9 years of age, these germs would already be visible radiographically, and for this reason the authors of this report also affirmed that the child presented absences of these teeth.

The inevitable consequences of oligodontias include malocclusions due to the inadequate position of the teeth during growth, deficiency of the alveolar processes due to the lack of teeth, and excess of spaces between the dental arches [32]. In the present case, all the abovementioned characteristics were evident, so that this patient was indicated for orthodontic treatment. Moreover, at an appropriate age, this patient will also be prosthetically rehabilitated.

In summary, tooth ageneses constitute a clinical and public health problem, because patients in these conditions may suffer a reduction in their masticatory capacity, malocclusions, phonoaudiological problems, and compromised esthetics. These problems may also affect the behavioral pattern and social life of these persons, so that early diagnosis and guidance with regard to treatment are necessary, thus being a condition that pediatric dentists must be capable of diagnosing.

## Figures and Tables

**Table 1:** Candidate gene markers studied.

Gene and base change	Location in the gene	SNP	Functional consequence	Locus	References
*MMP3* (**A**/G)	promoter	rs522616	Upstream variant 2 KB	11q22.2	Letra et al. [15, 16]
*BMP2* (**C**/T)	unknown	rs1884302	downstream gene variant	20p12.3	Justice et al. [13]

Note: bold forms indicate wild allele, obtained from databases: http://www.ncbi.nlm.nih.gov and http://genome.ucsc.edu.
